# *ALEPH*: a network-oriented approach for the generation of fragment-based libraries and for structure interpretation

**DOI:** 10.1107/S2059798320001679

**Published:** 2020-02-26

**Authors:** Ana Medina, Josep Triviño, Rafael J. Borges, Claudia Millán, Isabel Usón, Massimo D. Sammito

**Affiliations:** a Crystallographic Methods, Institute of Molecular Biology of Barcelona (IBMB–CSIC), Barcelona Science Park, Helix Building, Baldiri Reixac 15, 08028 Barcelona, Spain; b Departamento de Física e Biofísica, Instituto de Biociências, Universidade Estadual Paulista (UNESP), Botucatu-SP 18618-689, Brazil; c ICREA, Institució Catalana de Recerca i Estudis Avançats, Passeig Lluís Companys 23, 08003 Barcelona, Spain; d Department of Haematology, Cambridge Institute for Medical Research, University of Cambridge, Hills Road, Cambridge CB2 0XY, England

**Keywords:** X-ray phasing, bioinformatics, folding, characteristic vector, fold clustering, *ALEPH*, fragment-based libraries

## Abstract

The analysis of large structural databases reveals general features and relationships among proteins, providing useful insight. A different approach is required to characterize ubiquitous secondary-structure elements, where flexibility is essential in order to capture small local differences. The *ALEPH* software is optimized for the analysis and the extraction of small protein folds by relying on their geometry rather than on their sequence. The annotation of the structural variability of a given fold provides valuable information for fragment-based molecular-replacement methods, in which testing alternative model hypotheses can succeed in solving difficult structures when no homology models are available or are successful. *ARCIMBOLDO_BORGES* combines the use of composite secondary-structure elements as a search model with density modification and tracing to reveal the rest of the structure when both steps are successful. This phasing method relies on general fold libraries describing variations around a given pattern of β-sheets and helices extracted using *ALEPH*. The program introduces characteristic vectors defined from the main-chain atoms as a way to describe the geometrical properties of the structure. *ALEPH* encodes structural properties in a graph network, the exploration of which allows secondary-structure annotation, decomposition of a structure into small compact folds, generation of libraries of models representing a variation of a given fold and finally superposition of these folds onto a target structure. These functions are available through a graphical interface designed to interactively show the results of structure manipulation, annotation, fold decomposition, clustering and library generation. *ALEPH* can produce pictures of the graphs, structures and folds for publication purposes.

## Abstract 2

*ALEPH* characterizes the main-chain geometry of small, noncontinuous fragments to flexibly annotate secondary structure, decompose folds, extract libraries and superpose fragments. Secondary and tertiary structure are described through networks of characteristic vectors, which are defined between the centroids of the C^α^ and carbonyl O atoms in a peptide.

## 1. Introduction   

Secondary-structure properties are usually derived from the hydrogen-bond pattern. They were predicted even before the structures of full proteins had been determined (Pauling *et al.*, 1951 ▸; Pauling & Corey, 1951 ▸). Analysing this network implies assessment of the environment of the amino acid in a peptide, made up of nonconsecutive residues, which may encompass symmetry equivalents that are not explicitly contained in the PDB set of coordinates. The formation of these hydrogen bonds and the planarity of the peptide bond restrict the protein backbone to adopting torsion-angle values in characteristic ranges, corresponding to the most populated areas of the Ramachandran plot (Ramachandran *et al.*, 1963 ▸). Conversely, the analysis of the relevant torsion angles may suffice to characterize the secondary structure. *Definition of Secondary Structure of Protein* (*DSSP*) is the standard algorithm employed for the prediction of hydrogen positions and bonds, from which the secondary-structure environment for each residue can be derived (Kabsch & Sander, 1983 ▸; Touw *et al.*, 2015 ▸). Distortions in the polypeptide chain are sometimes encountered, and especially when the resolution falls below 3–3.5 Å (Headd *et al.*, 2012 ▸; Karmali *et al.*, 2009 ▸) some structures may fail to meet *DSSP* regularity. *DipSpace* (Pereira & Lamzin, 2017 ▸) embeds geometrical information about the backbone atoms around each C^α^ atom in its dipeptide-unit environment, which is described as a matrix of the interatomic distances. Also, *CaBLAM* (Richardson *et al.*, 2018 ▸) defines a novel parameter space of C^α^–C^α^ and CO–CO virtual dihedrals, where the CO dimension diagnoses large distortions of peptide orientation at low resolution and the two C^α^ dimensions identify the probable secondary structure obscured by these problems. *CaBLAM* is designed for structure validation to detect errors in the model, whereby poor geometry introduces ambiguity.

For our purposes, further abstraction can be achieved by focusing on the carbonyl bond as a lever in the necessary torsions to form hydrogen bonds. This gives rise to a characteristic atomic distribution within archetypal secondary-structure elements (Sammito *et al.*, 2013 ▸). We denominate the vectors defined from the centroids of all α-carbons to the centroids of all carbonyl O atoms in a polypeptide stretch as ‘characteristic vectors’ (CVs). Beyond the description of secondary structure, such vectors can be used to characterize the fold through their relative angles and distances. Also, locating them in a spatial context makes geometrical comparisons possible. The advantage of CVs is that the same reduction in dimensionality can be applied within different scopes: for example, the environment of single amino acids, when CVs are calculated over overlapping tripeptides, or to secondary-structure units in a fold, when CVs are defined over such longer stretches. This formalism is particularly useful for the geometric description of the small fragments used for phasing in the *ARCIMBOLDO* programs (Millán *et al.*, 2015 ▸). Since the first implementation of the method (Rodríguez *et al.*, 2009 ▸), combining molecular-replacement (MR) searches of small secondary-structure fragments with *Phaser* (McCoy *et al.*, 2007 ▸) and density modification and autotracing with *SHELXE* (Sheldrick, 2010 ▸), *ARCIMBOLDO* has been extended to integrate other sources of information (Rodríguez *et al.*, 2012 ▸) and diversified to use libraries of fragments (Sammito *et al.*, 2013 ▸). Several bioinformatics tools are available to extract folds or models similar to a template structure using sequence or structural alignments. The *Dali* server (Holm, 2019 ▸) is a web service from which the user can obtain a sorted hit list corresponding to a specific input fold, *MASTER* (Zhou & Grigoryan, 2015 ▸) defines a new r.m.s.d.-based metric to explore and extract fragments from a precomputed database and *PDBeFold* (Krissinel & Henrick, 2005 ▸) is based on multiple structure alignments across families of structures. Our approach, *ALEPH*, is designed for customizable use with small fragments. We combine the definition of new geo­metrical descriptors, such as CVs, with network algorithms to address fundamentally different questions. The user can control the desired strictness to accurately extract very specialized secondary-structure elements as well as general ubiquitous folds. In many applications, such a level of flexibility is fundamental to draw conclusions for different structural questions. Fragment-based MR, for example, requires a finer sampling of fold variations. In fact, characteristic vectors can be defined over shorter or longer stretches to capture fine or coarse features.

Suitable fragments from distant homologs can also be identified (Sammito *et al.*, 2014 ▸) or improved (Millán *et al.*, 2018 ▸) against the experimental data. CVs are used in all operations involved in identifying, extracting, comparing and annotating fragments to refine subsequent degrees of freedom (McCoy *et al.*, 2018 ▸). CVs are also used in the verification step introduced to establish the correctness of coiled-coil solutions at low resolution (Caballero *et al.*, 2018 ▸). Finally, we use them in the analysis of solved cases for development purposes. As phasing methods using small fragments are becoming very popular in successful pipelines such as *AMPLE* (Bibby *et al.*, 2012 ▸), *Fragon* (Jenkins, 2018 ▸) and *FRAP* (Shrestha & Zhang, 2015 ▸), and other *ab initio* approaches to phasing such as *I-TASSER* (Roy *et al.*, 2010 ▸) and *MR-Rosetta* (DiMaio *et al.*, 2011 ▸), CVs might find use in this context, where accurate structural characterization independent of the sequence is needed.

Here, we present the CV-based program *ALEPH*, which was developed as a bioinformatics tool to handle fragments and prepare libraries representing variations of a given fold for MR. Extraction of such libraries is performed without relying on sequences or alignments to allow searches across different families.

## 2. Materials and methods   

### 2.1. Software versions   

*ALEPH* is written in Python 3, requiring 3.7+. The code is developed to maintain retro-compatibility with Python 2.7, although the use of a Python 3 interpreter is strongly advised whenever possible. Tutorials and documentation are available from our website (http://chango.ibmb.csic.es/ALEPH). The graphical user interface is written in Python 3 with Pyside2 and QT5. Python libraries and environment variables are managed through Conda (https://anaconda.org).

*ALEPH* requires the libraries listed in Table 1 ▸. *ALEPH* is distributed through PyPI (https://pypi.org/project/pip/). From a Python 3 (https://www.python.org/) environment, installation only requires execution of the command pip install aleph.

The command alephui launches the graphical interface. The core program is also available from the command line through the command aleph. Fragment-based MR tests have always been performed through the *ARCIMBOLDO* framework (Millán *et al.*, 2015 ▸), which relies on the *Phaser* intensity-based maximum-likelihood function rendering the log-likelihood gain score (Read & McCoy, 2016 ▸) in version 2.7 and upwards, and on the correlation coefficient between observed and calculated normalized intensities (Fujinaga & Read, 1987 ▸) as calculated in *SHELXE* (Sheldrick, 2002 ▸) version 2019. *Phaser* 2.8 was used through its *CCP*4 7.0 (Winn *et al.*, 2011 ▸) or *Phenix* 1.17 (Liebschner *et al.*, 2019 ▸) distributions. Structure-amplitude-weighted mean phase errors (wMPEs; Lunin & Woolfson, 1993 ▸) were calculated with *SHELXE* against the models available from the PDB to assess performance. The model and maps were examined with *Coot* 0.8.9.1 (Emsley *et al.*, 2010 ▸). The figures were prepared with *PyMOL* 2.2.0 (Schrödinger). *GEPHI* 0.9.2 (Bastian *et al.*, 2009 ▸) and the free version of *yEd* (https://www.yworks.com/) were used to read *xmlgraph* files and produce network pictures for this manuscript.

### 2.2. Computing setup   

Library-generation tests were run on a local HTCondor version 8.4.5 (Tannenbaum *et al.*, 2001 ▸) grid made up of 160 nodes totalling 225 Gflops. Some libraries were generated on a single workstation with two Intel Xeon E5-2680 processors totalling 24 physical cores and 128 GB RAM running Ubuntu Linux. Typical running times for library generation from the whole PDB ranged from 6 to 12 h on a single workstation of 24 cores. Times vary substantially depending on the nature of the fold and on the dedicated hardware. Smaller folds tend to be more general and require more computation to process the vast number of occurrences that are found. The database used for extraction may be filtered or limited to accelerate the process. Parameterization is also key: more lax, lower thresholds in the geometrical similarity to the template will increase the number of fragments to process and hence the time. Often the library produced is over-sampled for phasing purposes and needs to be clustered to eliminate redundancy. This process of reducing millions of models to tens of thousands can take one to three days.

## 3. *ALEPH* as a composite bioinformatics tool   

Recent developments in MR have formally bound the solvability of the phase problem to an estimated LLG (eLLG; McCoy *et al.*, 2017 ▸), allowing the minimum fractional scattering that is needed at a given accuracy to be established *a priori* (Oeffner *et al.*, 2013 ▸). The eLLG score is used in the fragment-based MR approach *ARCIMBOLDO* to guide the difficult trade-off between fragment generality and solution discrimination (Oeffner *et al.*, 2018 ▸). While minimal fragments, such as simple secondary-structure elements, are ubiquitous across structures, their correct location usually renders a low signal. Small local folds, defined as composite sets of discontinuous secondary-structure elements (for example, three antiparallel β-strands facing two parallel helices), are still ubiquitous across different families of structures but, unlike α-helices, cannot be represented accurately enough through a single model that will match the corresponding geometry in most unknown target structures. In this context, we developed *ALEPH* as a bioinformatics tool to prepare libraries representing variations of a given fold for MR. The extraction of such libraries is performed without relying on sequences and alignments to allow searches across different families.

**Figure 1:**
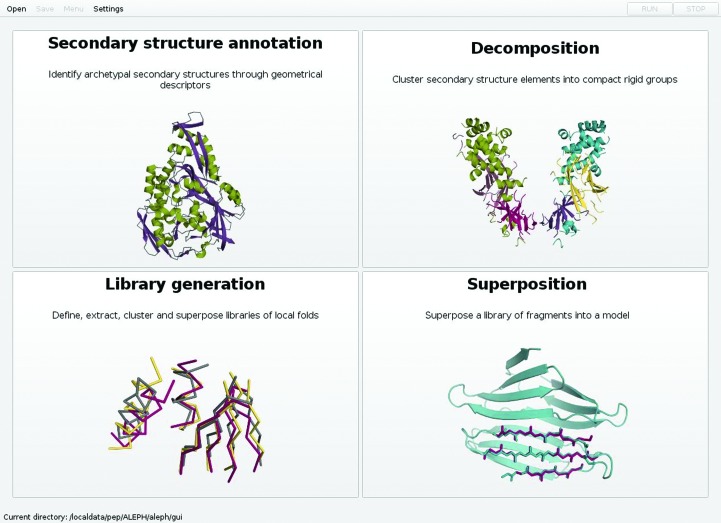
Main menu in the graphical user interface accessing the four functions in *ALEPH*: secondary-structure annotation with graphs, fold decomposition through community clustering, template-based library generation and superposition of small fragments.

*ALEPH* provides a convenient graphical user interface to perform four different tasks: flexible secondary-structure and tertiary-structure annotation, mapping any protein structure into a network, decomposing a structure into smaller local folds, and generating customized libraries of local folds and superposing small fragments onto complete protein structures. Fig. 1 ▸ displays the main menu of the graphical interface used to access these tasks.

*ALEPH* performs four clearly differentiated tasks. The annotation mode writes the annotated secondary-structure elements in a PDB file and plots of the graphs describing the geometrical properties of the CVs (as PNG files). The decomposition outputs a PDB file with a different chain identifier for each group. These coordinate files are ready to be used by *ARCIMBOLDO* or *Phaser* to perform *gyre* and *gimble* refinement of the model (McCoy *et al.*, 2018 ▸). The library generation places all extracted folds superposed on the reference template in a new directory library. If clustering is performed, an additional clusters directory is output. The superposition mode writes the PDB file of the superposed target structure. Any *ALEPH* run collects all of the output needed for the graphical interface to show the results in running time. The format of this file is standard JSON so it can be inspected programmatically.

### 3.1. Secondary- and tertiary-structure annotation   

All algorithms in *ALEPH* rely on the geometrical representation of the main chain of a protein using a discrete distribution of CVs. Originally introduced in the first implementation of *ARCIMBOLDO_BORGES* (Sammito *et al.*, 2013 ▸), *ALEPH* recasts their use in networks. For any peptide of at least three residues, a CV is uniquely identified as the vector connecting the geometric centroid of all C^α^ atoms to the centroid of the O involved. The main chain is annotated for all possible tripeptides with an overlapping window of one residue. These vectors provide a smoothed description of the protein backbone, revealing local main-chain distortions from an idealized secondary structure. Each residue is then associated with at least one CV and is annotated with the probability of its being part of a helix, a strand or a coil region. The relationships between CVs are described with a complete graph network.

While a single secondary-structure annotation may suffice for general purposes, we have encountered the need to control the strictness with which we want to query secondary structure and the need to formulate the alternative questions: ‘From which secondary structure is a distorted fragment derived?’ or ‘How close is one local fold to another?’ In the context of fragment-based MR, such questions underlie decisions on how to extract or decompose models and which degrees of freedom to confer. Thus, when defining the local geometry and conformation of a small local fold it might be desirable to explore different annotations. Our aim in *ALEPH* is to provide real-time, graphical control over different levels of annotation, smoothly relaxing restraints to ideal fragments and allowing the interactive tailoring of parameterization to a particular purpose.

#### 3.1.1. Implementation   

**Figure 2:**
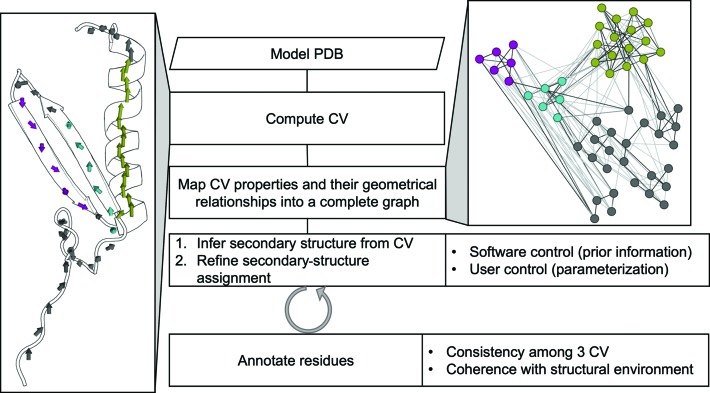
*ALEPH* workflow for the annotation mode.

The annotation algorithm starts from the computation of CVs and proceeds to the iterative interpretation of their secondary and tertiary structures. The algorithm ends with the unequivocal association of a secondary-structure type, or coil, with each residue in the main chain. The general workflow is shown in Fig. 2 ▸. *ALEPH* maps main-chain structure into a mathematical model using as generic geo­metrical descriptors overlapping CVs generated with a window size of one residue. Not only secondary structure but also coils or conserved loops can be queried and compared (Pröpper *et al.*, 2014 ▸). The geometrical relationships among these vectors are stored in a sparse matrix that can be compared against similarly annotated matrices to extract local folds.

A structure is input through a standard PDB format file. The Biopython library is used to validate the format and parse the sequence, coordinates, occupancies and *B* factors from the PDB file. No secondary-structure annotation is imported. Filtering on occupancy reduces disordered residues to a single conformation and only residues containing all main-chain atoms are stored. Water molecules are also discarded. Connectivity between residues relies on a distance test between N and C atoms rather than on residue identifiers. For a stretch of polypeptide chain, a CV is defined with its origin at the centroid of all of its C^α^ atoms and its end at the centroid of all carbonyl O atoms. A minimum number of three residues is needed to describe secondary-structure features. To determine whether this minimum number was also the optimal number, a statistical analysis was conducted against a pool of 18 646 structures determined by X-ray crystallography to resolutions of 2.1 Å or better from the PDB filtered at 90% sequence identity. From these models, several sets of non-overlapping CVs were computed. In each set, CVs were generated from a different number of residues: three, nine, 15 and 21. All CV moduli were correlated to the standard *DSSP* annotation (Kabsch & Sander, 1983 ▸). The analysis showed two distributions centred at two different means: 1.4 Å for β-strands and 2.2 Å for α-helices. A Kruskal–Wallis equality of populations rank for comparing the medians of each data set revealed a significant difference (Kruskal & Wallis, 1952 ▸). Indeed, large fragments tend to bend far away from the ideal description of an α-helix or β-strand. Moreover, we could establish that the angle between consecutive vectors belonging to an α-helix varies from 5 to 10°, whereas the range is from 50 to 55° for consecutive vectors in a β-strand. Once again, the ranges are separate enough to avoid ambiguity.

The use of a single CV to capture the entire geometrical property of a secondary-structure element, as previously proposed (Sammito *et al.*, 2013 ▸), was already sufficient to extract folds similar to a given template and generate libraries for fragment phasing. However, the new approach makes these vectors more comparable across different structures. As seen from our analysis, the CV distribution of tripeptides for each secondary-structure type presents small standard deviation, high kurtosis and low skewness. Curvature and bending inside a fragment are instead described by the moduli variations over the main chain observed in the discrete overlapping distribution.

The annotation algorithm in *ALEPH* maps the distribution of overlapping CVs into a complete undirected edge-weighted graph, where a node represents a CV and an edge connecting two nodes stores the angle between the two connected CVs and their Euclidean distance. Each CV is assigned to an α-helix (*ah*), β-strand (*bs*) or coil (*coil*) region by evaluating a penalty function, in which geometrical descriptors are used to determine the distance score of the CV from an ideal helix or strand. To evaluate the structural environment, the algorithm also includes distances and angles across different fragments. This pseudo-distance function has been heuristically estimated from the analysis of pre-annotated secondary-structure vectors. If the absolute difference between the two scores is larger than a chosen threshold that we call ‘strictness’, then the CV is annotated according to the lowest score. Otherwise, it will be annotated as a coil. The procedure involves several iterations in which the algorithm refines the weights and the values of each descriptor, improving the analysis of the structural environment.

The result at this point is an annotation for CVs, as each residue can participate in up to three different CVs. Translation into a residue annotation follows three rules.

(i) If all CVs in which a residue participates are annotated as either *ah* or *bs*, so is the residue.
(ii) If two of the CVs in which a residue participates are annotated as *ah* and none as *bs*, and the following residue is annotated as *ah*, then the current residue is annotated as *ah*.
(iii) A residue originally marked as *coil* will finally be annotated as *bs* if it participates in two CVs annotated as *bs* and none as *ah*, or if it participates in at least one CV annotated as *bs* and one of the next or previous two residues is annotated as *coil*.

The last two rules are introduced to assign terminal residues in fragments separated by a short span of coil.

Once secondary-structure fragments have been annotated, their spatial relationship is annotated by mapping fragments onto a new graph where each fragment is represented by a supernode gathering all of its CV nodes. Edges relating these supernodes are annotated to describe their spatial relationships with the minimum, maximum and average of all angles and distances.

Edges are weighted by the inverse of the average distance and multiplied by a constant factor if the secondary structures connected are of the same type. In this way, fragments close in space will be related by higher weights and packing of β-sheets will be promoted. Edge weights prove useful for structure decomposition, as described in Section 3.2.1. Concomitantly, β-strands are packed into β-sheets and annotated in groups. Two strands will belong to the same sheet if at least 40% of the CV angles between the two fragments follow the empirical distribution observed for parallel and antiparallel β-strand CVs and their distance is lower than 6 Å.

Plots of the discrete distribution of CV moduli, plots of angles between consecutive CVs and plots of C^α^–C^α^ distances are generated, together with the corresponding tables reporting all numerical values. These plots can be used to identify anomalies, spot errors in the main chain and evaluate the goodness of the annotation, and are displayed in the graphical interface.

#### 3.1.2. Examples: secondary-structure annotation for OppA and two helices of photosystem I   

**Figure 3:**
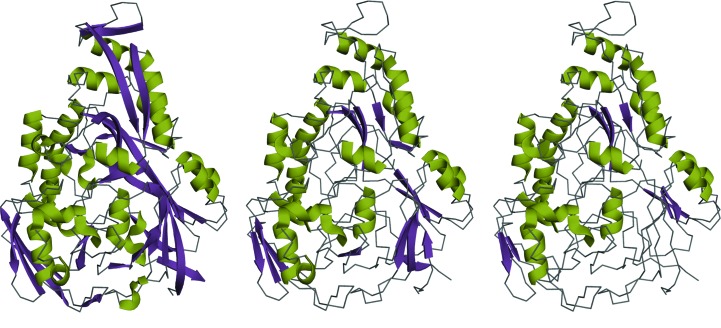
Comparison of the *ALEPH* annotation for PDB entry 1xoc with different parameterizations: strictness for α-helices and β-strands set to (*a*) 0.2, (*b*) 0.55 and (*c*) 0.6. The percentage of residues annotated as α-helix and β-strand fragments are (*a*) 33% and 33%, (*b*) 28% and 15% and (*c*) 28% and 7%, respectively. As the strictness threshold is increased, the algorithm annotates shorter, more ideal fragments. Colours represent secondary-structure types: green for α-helices and purple for β-strands.

The structure of the peptide-binding protein OppA in complex with an endogenous peptide (PDB entry 1xoc) contains one chain of 520 amino acids and a peptide of nine amino acids (Levdikov *et al.*, 2005 ▸). The space group is *P*2_1_2_1_2 and the resolution is 1.55 Å. The α/β structure encompasses three domains according to the CATH server. *DSSP* annotates 29% helical residues and 23% β-sheet. *ALEPH* annotations of PDB entry 1xoc considering different strictness thresholds are shown in in Fig. 3 ▸. Selecting a lower strictness allows secondary-structure elements to be extended, especially β-strands approximating bent fragments (Fig. 3 ▸ *c*). In the case of PDB entry 1xoc a strictness of 0.55 (Fig. 3 ▸ *b*) or higher will produce annotations that maintain the hydrogen-bond patterns establishing secondary-structure elements. At the lowest threshold of 0.2 (Fig. 3 ▸ *a*) the hydrogen-bond pattern is occasionally broken; one residue is even found outside the secondary-structure area of the Ramachandran plot, while four residues are on the limit. More precise annotation can be found at higher strictnesses where the fragments tend to be shorter, accumulating less curvature. Depending on the intended application, one or other of the annotations might be preferred.

**Figure 4:**
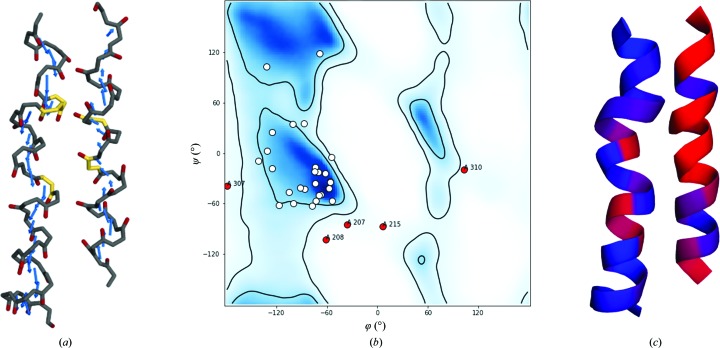
Annotation of severely distorted helices. (*a*) Main-chain backbone representation of the distorted helices from chain *A* of photosystem I (PDB entry 2o01); carbonyl O atoms are coloured red. CVs are represented by blue arrows and the Ramachandran outliers are highlighted in yellow (amino acids 207, 208, 215, 307 and 310); they are displayed as red dots in the Ramachandran plot (*b*). (*c*) The strictness is annotated for each residue: blue indicates higher confidence than red.

One example in which a less stringent description of the overall fold is preferable is the annotation of secondary-structure elements that present serious errors in the main chain. Although the fragment is an incorrect physical model, its approximation to a secondary-structure element can support the identification and correction of errors. Two fragments can be annotated as distorted helices (Fig. 4 ▸ *a*): amino acids 201–227 and 298–317 from chain *A* of the photosystem I supercomplex (PDB entry 2o01; Amunts *et al.*, 2007 ▸). The direction of the carbonyl bonds is not parallel to the helical axis and this is reflected by shorter CV moduli and larger deviations in the angles between consecutive CVs than those found in α-helices. The distances of consecutive C^α^ atoms in the fragments are not constant.

The Ramachandran plot presents several outliers (Fig. 4 ▸ *b*), which correspond to poor CV scores for α-helices in the annotation. In the annotation procedure *ALEPH* produces a file called strictnesses.pdb, which is displayed in the graphical interface. It shows the maximum strictness threshold required to annotate each residue as part of a secondary-structure element. Small values (red) in the difference between the scores for an α-helix hypothesis and a β-strand hypothesis imply low confidence in the annotation; conversely, a larger value (blue) indicates a clear discrimination.

Hence, from analysis of the strictnesses.pdb output and inspection of the two helices of interest (Fig. 4 ▸ *c*), we can observe large errors in both helices. Notice how CVs are sensitive to the misorientation of the carbonyl O atom and are less affected by a deformation of the helix turn.

Such poor geometry leads to differences in the annotations produced by *DSSP* and *CaBLAM*. Here, an analysis of the residue-based strictness output by *ALEPH* could be a useful tool to spot the general secondary-structure features, distorted helical conformation and the local regions of low confidence, and hence the poor geometry.

### 3.2. Decomposition through community clustering   

This section describes decomposition with *ALEPH* of given protein folds into rigid subparts that will allow the comparison of proteins with overall similar folds but local dissimilarities. Network community clustering constitutes a set of algorithms that distribute all nodes in the graph into non-overlapping groups to maximize the modularity score (Newman, 2006*b* ▸) of the graph. Formally, this score is defined as the fraction of the edges that fall within the given groups minus the expected fraction if the edges were distributed at random. Intuitively, it can be seen as a score that, if high, reflects dense connections between the nodes within groups but sparse connections between nodes in different groups. In the context of MR, this decomposition can be used for the identification of compact rigid groups to refine their relative rotation and translation with respect to the other groups. It is known that protein domains or smaller motifs across homologous structures can move concertedly with respect to the overall fold; thus, even for pairs of structures sharing a very high sequence identity (above 60%) it is common to observe deviations derived from conformational flexibility. Allowing the model additional degrees of freedom results in an increase in signal, enhancing the discrimination of the correct solution, improving the density map and providing a better partial solution for an eventual further search. The annotation of these groups with *ALEPH* is used in the spherical mode of *ARCIMBOLDO_SHREDDER* (Millán *et al.*, 2018 ▸).

#### 3.2.1. Implementation   

Decomposition of a structure into compact folds is achieved by generating a graph in which each node represents a single secondary-structure element and the edges store statistical properties reflecting the geometrical relationship between the fragments. In particular, an average distance between two fragments is defined as the mean distance among all of the CVs involved in the pair. This number is used as the weight employed by the community clustering algorithm to optimize the group classification. Although not directly corresponding to a physical property of the two fragments, it is a measure of proximity and allows the algorithms to generate compact folds.

The algorithm can force clustering to respect structural constraints, encouraging the formation of groups. For example, it is useful to cluster together the β-strands in a sheet. The decomposition algorithm optimizes the modularity score of the graph but can be biased to promote the formation of size-homogenous clusters containing the same number of secondary-structure elements, as discussed in Appendix *A* [App appa]. These constraints are controlled by the edge weights in the graph. *ALEPH* also provides a hierarchical decomposition in which the clustering procedure is iterated, increasing the number of groups to be output. This method generates a dendrogram in which each level corresponds to a progressive decomposition, ranging from all of the secondary-structure elements being included in one single cluster to each secondary-structure element belonging to a separate cluster. *ALEPH* graphically represents the dendrogram and the hierarchical structural decomposition, opening a route to structural interpretations of the fold classification.

**Figure 5:**
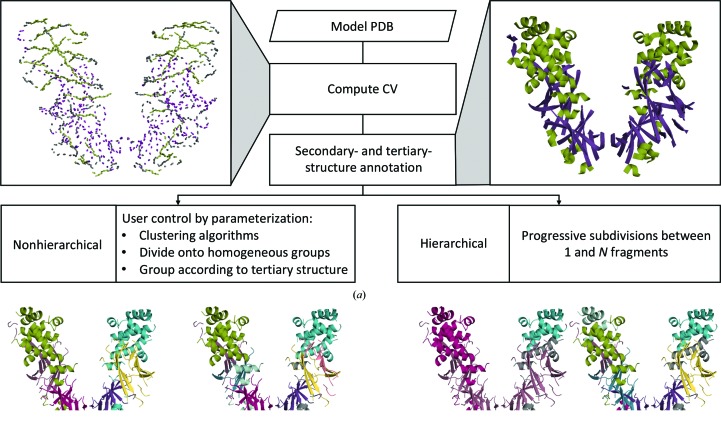
Workflow for the decomposition algorithm exemplified using the diphtheria toxin. (*a*) The program can alternatively perform a hierarchical or a nonhierarchical clustering. (*b*) Structural constraints are used, forming homogenous clusters and forcing β-strands to pack into β-sheets. (*c*, *d*) Alternative decompositions performed (*c*) with no constraints and (*d*) hierarchically.

The workflow of the algorithm is shown in Fig. 5 ▸, illustrating the decomposition of the dimer formed by the wild-type diphtheria toxin (PDB entry 1f0l) as discussed in the next section.

#### 3.2.2. Example: decomposition of the wild-type diphtheria toxin   

The diphtheria toxin from corynephage beta (PDB entry 1f0l) is an ADP-ribosyltransferase which inhibits eukaryotic protein synthesis by inactivating elongation factor 2. The crystal structure, which was determined to 1.55 Å resolution in space group *P*2_1_2_1_2, contains a homodimer. Each monomer is composed of 535 residues divided into three different domains, each belonging to a different superfamily: an N-terminal α–β complex, a central immunoglobulin-like domain and a C-terminal helical orthogonal bundle domain with globin-like topology.

Decompositions of the structure with different parameterizations were carried out to reveal the structural groups of the protein (Fig. 5 ▸). A nonhierarchical clustering, constraining groups to have a homogenous size and forcing strands to pack in β-sheets within the same cluster, resulted in a more biologically sensible classification, reflecting the three domains described above (Figs. 5 ▸ *b* and 5 ▸ *c*). On the other hand, a hierarchical clustering in which the sub-decomposition was performed sequentially revealed different levels of compactness from the formation of the dimer to the nearest-neighbour fragment (Fig. 5 ▸ *d*).

### 3.3. Library generation   

In the context of fragment-based MR, the generation of a set of models representing the same small local fold may be used. Sequence-derived libraries from *Rosetta* are used in *ab initio* models for phasing (Rigden *et al.*, 2008 ▸). Such libraries provide sparse building blocks to approximately cover any part of a structure, whereas our libraries of superimposed models represent variations of a given geometry to find an accurate fragment. Previous knowledge can be used to filter the PDB and select the subset of structures from which to extract the library. The sequence-free extraction method is particularly useful for small and general folds that are ubiquitous in different protein families.

The generation of a library comprises five steps.

Step 1. Define the local folds to be extracted through a PDB template and select the parameterization.
Step 2. Parse and annotate the proteins stored as PDB files within a given directory or download a subset of structures based on a sequence or a family. Optionally, filter.
Step 3. Extract from the set of proteins every occurrence of the local fold, comparing and filtering with customizable thresholds.
Step 4. Superpose models to the original template and save to file, setting a common *B* factor for all atoms.
Step 5. Cluster extracted models into geometrically similar groups.

It is possible to pre-annotate the whole PDB to speed up the procedure. Alternatively, the program annotates proteins during run time while executing a specific local fold search.

#### 3.3.1. Implementation   

**Figure 6:**
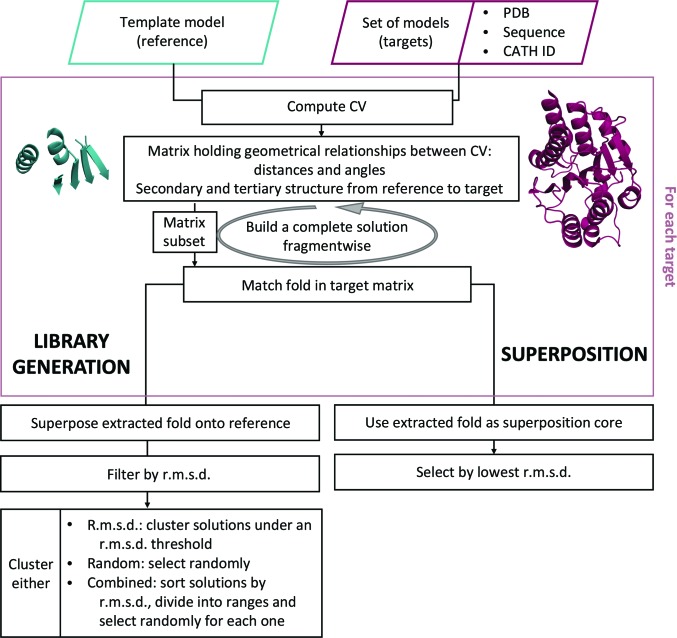
Library-extraction and superposition workflow. The minimal input required is a template in PDB format, which describes the fold to be extracted and the path to the stored PDB (or a subset database). For any other parameter a default is available, but the user might find it useful to adjust the strictness thresholds affecting the annotation of secondary-structure elements in both the template and the target and other parameters such as the intra-score and inter-score thresholds.

A library generator has previously been introduced (Sammito *et al.*, 2013 ▸), in which an entire secondary-structure fragment was mapped by a single CV. The length of each CV was used to annotate the secondary-structure element and to perform extractions based on relative geometrical properties. This initial implementation was already able to grasp the general properties of fragments and local folds, allowing the extraction of libraries for the solution of unknown structures. The simplification of the geometrical properties to one CV per secondary-structure element did not allow the fine control that has now been achieved. In the current implementation (Fig. 6 ▸), the algorithm has evolved to enhance control through two types of vector relationships: angles and distances between vectors in the same fragment describe secondary structure, while those relating different fragments characterize the fold. The user can define different thresholds, expressed as percentages, for the two types of relationships. A higher threshold for secondary-structure vectors will restrict the extraction of models to contain geometrically closer fragments to those in the template input, for example avoiding the extraction of bent helices if the template provides straight helices. The tertiary-structure parameter controls the similarity in the arrangement of the fragments into a fold: the higher the threshold, the closer the relative distances and angles.

Once the template model (in PDB format) is annotated with CVs the fold is searched against the whole PDB (or any set of structures given in a folder). The user can limit this search, providing a CATH family (Dawson *et al.*, 2017 ▸) or a *FASTA* sequence, which is used to perform a *BLAST* search against the PDB (https://www.rcsb.org/pages/webservices/rest-search), sorting the results by *E*-value and retrieving the SCOP (Murzin *et al.*, 1995 ▸) and CATH family from a candidate homologous structure with a minimum *E*-value of 0.005. The list of unique SCOP and CATH identities is then used to filter the database during the search.

The structures in the database to be queried are annotated with CVs. The search is then performed in parallel, distributing computation over a grid network or a supercomputer facility, or just by multiprocessing on a single workstation. By default, if the target structure contains several equivalent monomers, only one will be evaluated. As folds can also be formed requiring the participation of two or more different chains (for example a coiled coil), this parameter can be changed if so wished.

**Figure 7:**
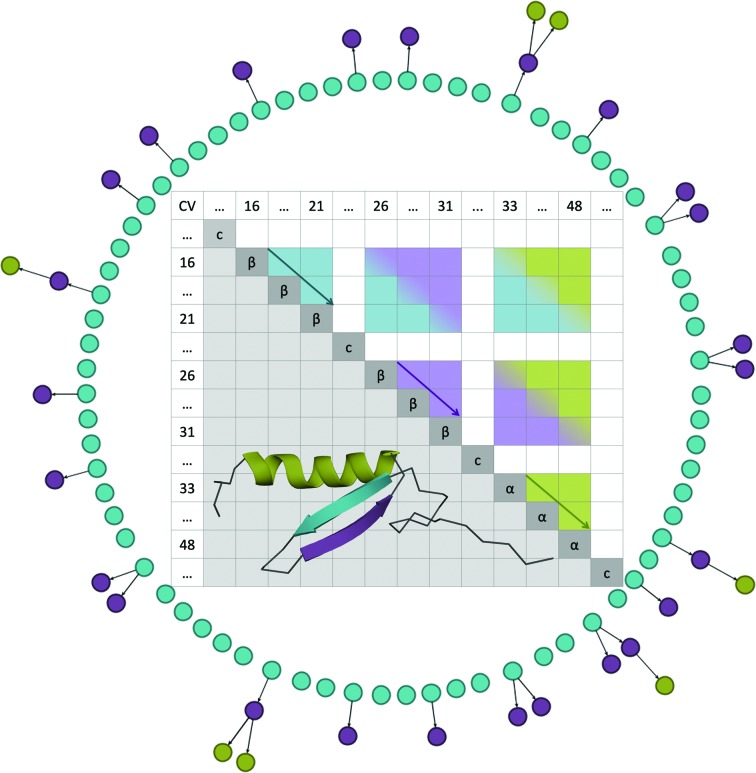
Scheme of the fold-extraction algorithm. The external graph represented in a circular layout is the forest of tree searches that are maintained in the memory by *ALEPH* during extraction from a target structure (PDB entry 3to7). Each layer of the circle is coloured with the same colour as the template fragment (from PDB entry 4e1p) to extract. The light-blue β-strand is the first fragment to be found, followed by the second, purple β-strand and finally by the helix. Leaves in the external layer represent solutions satisfying all geometrical constraints according to the thresholds set (60% for intra-vectors and inter-vectors). The half matrix displays the three fragments (compacting their lengths), showing triangle areas, coloured the same as the fragments, that carry the internal properties of each fragment and square areas where the geometrical relationships between two fragments are displayed using a colour gradient.

The graph resulting from the template annotation is stored as a matrix, in which the cell at (*i*, *j*) contains information about the angle and the distance between CV_*i*_ and CV_*j*_. Equivalent matrices are generated for every target structure in the database. The first diagonal would contain trivial self-relations, but is instead used to store the CV length and secondary-structure annotation. The second diagonal stores the relationships between contiguous vectors. Therefore, identifying similar secondary-structure elements, regardless of their relative orientations and distances, will only require exploring the second diagonal in a linear time computation. The extraction of a template-like fold, considering the possible secondary-structure fragments identified, will require analysis of the corresponding off-diagonal cells. Even if the chosen fold is present in the target structure, the composite fragments can be rearranged in a different order or be separated by insertions. The problem of searching compatible fragments in the second diagonal is solved recursively, as shown in Fig. 7 ▸, and the resulting submatrices in the template and target structure are compared with a distance. The successful extraction of a given fold, if present in an annotated protein, is guaranteed by the completeness of the CV network (any pair of nodes is connected by an edge). *ALEPH* has to find any coherent combination of fragments that simultaneously satisfies the template matrix, in which not all relations should be equally weighted; for example, angle differences in coil regions might be less strict than among secondary-structure elements.

The user can configure structural conditions: a sequence matching the template size can be provided. The symbol *X* indicates any valid residue. This parameter can be used to impose repeats or conserved cysteines. It is also possible to check for specific distances between S^γ^ atoms to enforce the presence of a disulfide bridge. Extracted models can be required to share the connectivity of the template, respecting the same N-terminal to C-terminal order in the fragments.

The extracted models are clustered into groups sharing closer geometry. This aims to reduce the number of models in the final library, avoiding redundant representation of the same variation of the fold. It also aims to better organize and discover fold properties, revealing the different types of observed conformation stored in the PDB. Alternative clustering algorithms are based on exhaustive pairwise comparison of r.m.s.d. between fragments, selection from an r.m.s.d. range to the template or a random selection of a subset of all possible occurrences. The choice should depend on the intended use of the library and the number of models to be extracted. While the first method does not involve a random selection, allowing reproducibility, and represents a finer criterion, it can have a long running time as the number of models extracted can be in the range of millions for very general ubiquitous folds. The other two methods are provided to perform faster clustering by sampling the space of the extracted models.

All of the models extracted and validated form a library that is superposed on the template and renamed according to the scheme pdbid_*x*_*yyyy*.pdb, where pdbid is the original identifier of the PDB structure from which the model has been extracted, *x* is the number of the structural model in the PDB entry (it differs from 0 only for NMR structures or ensemble models) and *yyyy* is an integer of a maximum of four digits that unequivocally identifies the model.

#### 3.3.2. Example: phasing NovP from *Streptomyces niveus* with a library   

To test the performance of the new library-generation algorithm in its use for phasing, we replicated our distributed libraries with fragments of the same secondary structure and generated new libraries of mixed α/β folds: ubiquitin-like and Rossmann folds. Here, we describe an example of an α/β-fold library used to phase the *O*-methyltransferase NovP from *S. niveus* (PDB entry 2wk1; García *et al.*, 2010 ▸). This protein is formed by a single monomer of 282 residues; the resolution of the data is 1.4 Å and the space group is *P*2.

**Figure 8:**
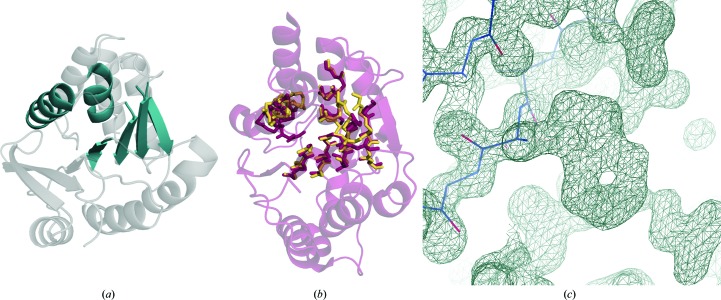
Solution of NovP with *ARCIMBOLDO_BORGES*. (*a*) Cartoon representation of the model, with PDB entry 1vid (grey) providing the template (turquoise) for library generation. (*b*) A model extracted from PDB entry 2gdz located by *Phaser* is shown in yellow and the final deposited structure of NovP (PDB entry 2wk1) is shown as a transparent red cartoon. (*c*) Detail of residue 119A: *F*_o_ FOM-weighted map at 3σ showing clear electron density for the side chain of a tryptophan after performing density modification and autotracing with *SHELXE*.

The model used to create the library was extracted from the catechol *O*-methyltransferase from *Rattus norveg­icus* (PDB entry 1vid; Vidgren *et al.*, 1994 ▸), showing a typical Rossmann-fold domain consisting of a central core of parallel β-strands with antiparallel α-helices on both sides. This fold is a very common fold found in many other protein families and thus is a good candidate for the generation of a general library of local protein folds. The particular fragment used as a template and shown in Fig. 8 ▸(*a*) contains four parallel β-strands and two α-helices on one side (amino acids 44–57, 60–65, 71–79, 84–90, 111–116 and 136–140 from chain *A* of PDB entry 1vid).

The data set used to generate the library is a subset of the PDB containing 18 349 X-ray models filtered at 90% sequence identity. We also removed structures deposited after the deposition of the test case (15 December 2009) to avoid bias in the results. We ran *ALEPH*, fixing strictness thresholds for α-helices and β-strands of 0.5 and 0.3, respectively. We set the secondary-structure score to 45% and the tertiary-structure score to 55%, allowing higher local variation within each fragment while restricting the overall fold more. We also imposed a maximum limit of a 5.0 Å r.m.s.d. to the template as a requirement to include models in the library. Clustering was not performed. The number of models that composed the superposed library output by *ALEPH* was 9413. The minimum r.m.s.d. obtained against the template was 0.2 Å for a model extracted from the template protein in a complex (PDB entry 3hvi). Library generation took approximately 9 h on a single workstation with eight cores. Some models were inspected and we could observe large rotations of the helices with respect to the β-sheet but preserving the distance from the plane defined by the helices to the β-sheet.

This library was used in *ARCIMBOLDO_BORGES* to phase the test protein PDB entry 2wk1. As the final refined structure of NovP was available, we could compute the wMPE of the output solutions and cluster phases in reciprocal space to count and identify the models from which the correct solution was found (Millán *et al.*, 2020 ▸). *ARCIMBOLDO_BORGES*, and hence the *Phaser* functions called, was run setting an initial r.m.s.d. of 0.6 Å. *gyre* refinement was skipped in the rotation step. After performing the translation search and packing check, the models were optimized with *gimble* refinement using the *ALEPH* annotation, defining three independent rigid blocks: two formed by each helix and the third for the β-sheet. 25 nonrandom solutions were found with a wMPE against the deposited structure ranging from 71.6° to 79.9°. All of them are related solutions, differing by less than 60° from one other. The solutions are achieved with models extracted from 21 different deposited structures: PDB entries 2igt, 2pbf, 2wdq, 3e9n, 1yde, 2yxe, 3bzb, 1ej0, 1spx, 2gdz, 1hxh, 1cyd, 1y5m, 1db3, 2hrb, 2b4q, 2nm0, 1o5i, 1xu9, 3ip1 and 2dm6. The sequence identities of these structures to NovP are practically negligible. PDB entry 2gdz, with a sequence identity of barely 5% and an overall r.m.s.d. of 7 Å to NovP, rendered the fragment providing the best solution. The original Rossmann fold cut from PDB entry 2gdz shows an r.m.s.d. of 3.1 Å. After decomposition and rigid-group refinement with *gimble* in *Phaser*, all of the β-strands and one helix were placed correctly and only one helix was still partially misplaced.

To extend the placed fragment to a complete solution, *SHELXE* was set to iterate 15 cycles of density modification and autotracing. The solvent content was set to 44%. The initial input model was trimmed to improve the correlation coefficient; in particular, *SHELXE* removed the misplaced helix. Data were extrapolated beyond the experimental resolution by up to 1 Å (Usón *et al.*, 2007 ▸). The new algorithm for tracing β-sheets in *SHELXE* was used to enhance tertiary-structure formation during tracing, as previously reported (Usón & Sheldrick, 2018 ▸). *ALEPH* has generated libraries from which the parameters for the new* SHELXE* tracing algorithm have been deduced.

After six cycles of autotracing, *ARCIMBOLDO_BORGES* output a definite solution with a model extracted from PDB entry 2gdz (Figs. 8 ▸ *b* and 8 ▸ *c*) that led to a *SHELXE* correlation coefficient of 34.9% with 199 residues traced. The completeness of the polypeptide trace was only 70%, as by default *ARCIMBOLDO* stops after identifying a clear solution (CC > 30) where model building can be completed by another program, such as *ARP*/*wARP* (Chojnowski *et al.*, 2020 ▸). In the electron-density map shown in Fig. 8 ▸(*c*), the side chains of aromatic amino acids are clearly visible. The initial r.m.s.d. of the extracted model belonging to the library to the final structure was 3.40 Å. After *gimble* refinement the model was improved, achieving a resolution of 2.18 Å, while the final r.m.s.d. after six cycles of tracing with *SHELXE* was 0.24 Å. Equivalent solutions were obtained from different structures presenting a lower initial r.m.s.d. to the target structure (for example a model from PDB entry 2pbf with an initial r.m.s.d. before *gimble* of 1.96 Å), but the *ARCIMBOLDO_BORGES* procedure stops as soon as a distinguishable solution is found and outputs the solution with the highest correlation coefficient.

### 3.4. Superposition   

Structural superposition is one of the most frequent tasks that is routinely performed during the analysis and interpretation of macromolecular structures. Several algorithms are in use, from those based on least-squares optimization of the root-mean-square deviations among a common set of atoms as in *LSQKAB* (Kabsch, 1976 ▸) to maximum-likelihood-based algorithms such as *THESEUS* (Theobald & Wuttke, 2006 ▸). A very fast algorithm based on dynamic programming is currently distributed through *CCP*4 under the name *GESAMT* (Krissinel, 2017 ▸), and molecular-modelling/visualization programs have implemented their own algorithms such as the secondary-structure matching in *Coot* (Emsley *et al.*, 2010 ▸) and sequence-based and sequence-free matching in *PyMOL* (Schrödinger). *TM-align* (Zhang & Skolnick, 2005 ▸) provides an alternative score encompassing the differences and extent of the match. *ALEPH* calculates this score for reference purposes, but its use in our context is limited.

Despite their high efficiency, these algorithms are optimized to superpose large connected domains or proteins and may sometimes run into difficulties when superposing small, disconnected folds or fragments onto a complete structure. For library generation in *ALEPH* we have developed a simple procedure to address this very specific task, which is made available through the graphical application. The use of the library-generation procedure to perform superposition is displayed in the workflow in Fig. 6 ▸.

#### 3.4.1. Implementation   

Most of the algorithms available to superpose protein structures differ in the selection of a common core. *ALEPH* uses the library-extraction algorithm to find every possible correspondence of the local fold to a target. From this point, the core, rotation and the translation that minimize the r.m.s.d. are optimized. Part of this procedure is to iteratively weight corresponding pairs of atoms to the inverse of the variance of the atom around the average structure (Nilges *et al.*, 1987 ▸) to improve the overall fit of the core. *ALEPH* allows additional trimming at the extremities of each secondary-structure element in the core. In particular, for each fragment with more than five residues, a maximum number of three residues can be removed from the extremities. All combinations are tested and used to calculate an r.m.s.d. The lowest r.m.s.d. will determine the best core and superposition to be output. If the local fold matches the target structure at multiple non-overlapping sites, *ALEPH* will output each of them separately. This feature may be useful to explore repetitions of a motif within a structure.

#### 3.4.2. Examples: superposition of small helical folds and β-stranded folds onto structures   

**Figure 9:**
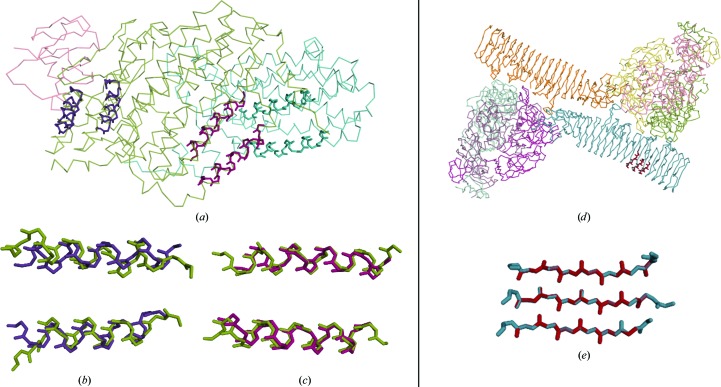
Example of the superposition of two small local folds onto complete structures. (*a*) Superposition by the *GESAMT* algorithm (purple) and the *SSM* algorithm (pink) of the library model and the protein with PDB entry 3n1z coloured by chains: chain *A*, orange; chain *B*, blue; chain *C*, green. The fragment contained in the library was extracted from the area represented as blue sticks. (*b*) Close-up view of the *GESAMT* superposition. (*c*) View of the *SSM* superposition. (*d*) Superposition between a model from PDB entry 2iou represented as red sticks and the whole structure represented as ribbons (coloured by chain) as determined by the *ALEPH* algorithm. (*e*) Stick representation of the superposed atoms.

As an example, we show the superposition of small α-helical and β-stranded fragments. A roto-translated model from a library of two parallel helices (Sammito *et al.*, 2013 ▸), extracted from the monooxygenase hydroxylase with PDB code 3n1z, was superposed against the whole structure as in Fig. 9 ▸(*a*). The superposition should be able to relocate the fragment in its original position. Algorithms that are not designed for small fragments may fail, especially when their main chain is disconnected. In the first example, both the *GESAMT* (Fig. 9 ▸ *b*) and *SSM* (Fig. 9 ▸ *c*) algorithms, through *SUPERPOSE* (Krissinel & Henrick, 2004 ▸), align fragments extracted from chain *B* onto chain *A* (with r.m.s.d.s of 2.02 and 0.75 Å, respectively). Chains *A* and *B* have different sizes and sequences and the identity between them is 17.9%. Superposition with *ALEPH* places the fragments exactly in their original location.

The second example tests a β-stranded local fold. Fig. 9 ▸(*d*) shows a ribbon representation of PDB entry 2iou. The complex is formed by three identical chains of major tropism determinant *P*1 along with a single chain of the pertactin domain, consisting of a large β-helix fold of 536 amino acids. A model extracted from PDB entry 2iou (Fig. 9 ▸ *e*) is contained in the three parallel β-strand library distributed through *CCP*4 (Winn *et al.*, 2011 ▸) with *ARCIMBOLDO_BORGES* (Sammito *et al.*, 2015 ▸). In this case we could not superpose the 20 amino-acid fragments using other methods, while *ALEPH* retrieved the correct superposition.

Finally, extraction of the library described in Sections 3.3.1 and 3.3.2 involves a superposition operation. According to *HHpred* (Zimmermann *et al.*, 2018 ▸), the PDB contained 126 homologs to our template structure, PDB entry 1vid, all with a low sequence identity ranging from 5% to 18%. *ALEPH* extracted library models from 61 (48%) of them: those where the secondary-structure elements defined in the template were present.

Finding the optimal superposition of a fragment onto another structure is a task that can be performed using other fast and sophisticated methods such as *GESAMT* and *SSM*. The examples reported here illustrate the application of our program to the challenging case of small, discontinuous fragments. Depending on the use, one or the other of the algorithms should be advantageous.

## 4. Distributed libraries   

Some of the libraries previously created with *ALEPH* are distributed with *CCP*4 for use as input search models in *ARCIMBOLDO_BORGES*. Recently, new libraries exploring more complex folds have been prepared and are available through our webpage. Table 2 ▸ lists the currently available libraries.

## 5. Conclusion   

This work introduces the new software *ALEPH*, a graph-based tool to annotate secondary and tertiary structure from coordinates, decompose a structure into compact local small folds, extract local folds from a database of structures without using the sequence and generate libraries of such folds, which are especially useful as input search models for fragment-based MR.

## Figures and Tables

**Table 1:** Summary of the Python libraries required by *ALEPH*

Library	Category	Reference
NumPy 1.16.2	Vectorized operations on matrices and vectors	Van der Walt *et al.* (2011 ▸)
Scikit-learn 0.20.3	Clustering and data mining	Pedregosa *et al.* (2012 ▸)
BioPython 1.73	Data handling of PDB files	Cock *et al.* (2009 ▸)
CSB 1.2.5	Maximum-likelihood-based superposition	Kalev *et al.* (2012 ▸)
Pyplot 3.0.3	Visualization of graphs and networks	Hunter (2007 ▸)
Python-igraph 0.7.1	Generation and management of networks in memory	Csardi & Nepusz (2006 ▸)

*ALEPH* uses the following community clustering algorithms		
Fastgreedy		Clauset *et al.* (2004 ▸)
Infomap		Rosvall & Bergstrom (2008 ▸)
Eigenvectors		Newman (2006*a* ▸)
Label propagation		Raghavan *et al.* (2007 ▸)
Community multilevel		Blondel *et al.* (2008 ▸)
Edge betweenness		Newman & Girvan (2004 ▸)
Spinglass		Reichardt & Bornholdt (2006 ▸)
Walktrap		Pons & Latapy (2005 ▸)

**Table 2:** Summary of the libraries distributed with the current version of *ALEPH* The internal nomenclature U (up) and D (down) is used to describe the relative orientations of the fragments composing the fold; thus, UUU means three parallel fragments and UDU means antiparallel. BS, β-strand; AH, α-helix.

	Fold	No. of template residues	Template PDB code	Template residues	No. of models	Novel structure solved
Helices	UD	34	3kfw	*X*163–179, *X*182–198	6343	4gdo
	UU	32	3rk2	*E*40–55, *H*157–172	11416	4gn0
Strands	UDU	20	4aeq	*B*66–71, *B*86–92, *B*96–102	7650	5ezu
	UUU	20	1c7e	*A*4–9, *A*52–58, *A*86–92	5844	
	UUD	20	4aeq	*A*22–27, *A*274–281, *A*313–318	7734	
	Sandwich BS UDU–UDU	43	4l1h	*A*18–24, *A*32–38, *A*61–67, *A*70–76, *A*83–90, *A*99–105	3069	
Combined	Ubiquitin BS UDDU + AH	39	1bt0	*A*1–7, *A*11–17, *A*23–34, *A*41–45, *A*64–71	3526	
	Rossmann BS UUUU + AH DD	47	1vid	*A*44–57, *A*60–65, *A*71–79, *A*84–90, *A*111–116, *A*136–140	9413	

## Appendix A

### A1. Cluster homogeneity in decomposition

The decomposition algorithm can be biased to promote the formation of homogenous clusters containing the same number of secondary-structure elements. This is achieved by performing a hierarchical decomposition, and for each iteration *k*, corresponding to a clustering in *k* groups, the modularity score of the decomposition graph is calculated as follows,

where *m* is the number of edges in the decomposition graph, **A** is the adjacency matrix of the graph, the elements *A*_*ij*_ of which are the total weight on edge (*i*, *j*), *k*_*i*_ is the total weight adjacent to node *i* and *k*_*j*_ is computed similarly. μ_*k*_ is the mean number of secondary-structure elements among all of the decomposed clusters and σ_*k*_ is the corresponding standard deviation. *N* is the number of secondary-structure elements in the whole structure and σ_*k*_/μ_*k*_ is the coefficient of variation. While this score promotes modularity, as previously defined (Newman, 2006*b* ▸), it is biased toward larger clusters with a low dispersion of elements and thus more homogenously sized groups.

### A2. Distance score between matrices in extraction

Some additional, more technical, details about the library-extraction algorithm are given here. Firstly, the graph resulting from the template annotation is stored as a set of two symmetric matrices in which each cell at (*i*, *j*) retains information about the angle and the distance between CV_*i*_ and CV_*j*_. The same pair of matrices are generated for every target structure in the database. *ALEPH* also initializes a weight matrix **W** that enhances the contribution from smaller fragments, distinguishing them from the noise of random hits.

The main properties of the matrices are as follows.

(i) They are symmetric, as distance lengths and minimum angles within pairs of vectors are commutative operations.
(ii) The first diagonal stores the coordinates for any CV and its modulus, the annotated secondary-structure type and the information relative to any tertiary-structure fold with which it has been previously associated.
(iii) The second diagonal stores the angles and the distances of each CV to the following CV. Secondary-structure elements remain defined by contiguous subsets of this diagonal. Two CVs are contiguous if the residues originating them are overlapping or contiguous.
(iv) Each secondary-structure element is uniquely referred to a set of matrix coordinates that enclose its CVs in the second diagonal.
(v) Identifying similar helices or strands, regardless of their relative orientations and distances, will only require exploring the second diagonal in a linear time computation.
(vi) Any cell that does not belong to the first diagonal stores the geometrical relationships between a specific pair of CVs; in particular, some of these cells store relationships among fragments.

A detailed description of the comparison between two matrices is now given describing the formulation of a distance score.

Suppose that *f*_1_, *f*_2_, …, *f*_*y*_ are fragments that have already been extracted and validated as one of the possible solution paths and *f*_*x*_ is the new fragment that should be added to the current solution. Each fragment *f*_*k*_ starts at the index s*f*_*k*_ and ends at the index e*f*_*k*_. Let **D**_r_ and **T**_r_ be the matrices (*n* × *n*) containing the distances and the angles of the CVs from the reference template, respectively, and **D**_t_ and **T**_t_ be the matrices for the distances and angles of the target structure from which fragments have been extracted. Then, let the new fragment *f*_*x*_ define a sub-upper triangular matrix **D**_r_(s*f_x_*, e*f_x_*; s*f_x_*, e*f_x_*); this matrix contains all of the intra-geometrical distances for the CVs contained in the fragment *f*_*i*_. Similarly, the sub-upper triangular matrix restricting **T**_r_ can be defined. Both matrices have their diagonal coincident with a subset of the diagonal of their original matrices **D**_r_ and **T**_r_. The objective is to extract all of the possible submatrices of the same size in the matrices **D**_t_ and **T**_t_, bounding their diagonals to be a subset of the **D**_t_ and **T**_t_ matrices.

The current extracted fragment *f*_*x*_ also defines two new matrices **F**_*xk*_ and **G**_*xk*_ that contains only the distances and the angles, respectively, between CVs belonging to fragments *f*_*x*_ and *f*_*k*_, which is any of the previously found and validated fragments. Every time a submatrix is extracted it has to be compared with the corresponding submatrix from the template to establish whether its geometrical parameters are similar enough and can be included in the current solution path.

The program establishes the following algorithm to calculate the difference between two matrices.

(i) Let **A** and **B** be two matrices of the same size (*n* × *n*) both containing distances or angles.
(ii) **A** and **B** have to be normalized. If they contain distances,
  If they contain angles,
(iii) A matrix difference *C* = *A_ij_* − *B_ij_* is computed.
(iv) The weighted mean and the weighted standard deviation of *C* are computed,
(v) Distance is then defined as *d* = 1.0 − [*C*_μ_ + (α*C*_σ_)] + ∊, where α = 1 if *n* ≥ 20, else α = 2, and ∊ = 0.1 if a Mann–Whitney test (Mann & Whitney, 1947 ▸) comparing **A** and **B** is true with a *p*-value of at least 0.1, else ∊ = −0.1.
